# Hyperglycemia-Induced Loss of Astrocytic Kir4.1 Is Associated with Increased DNMT1 Activity and Altered AMPK/SIRT1 Signaling

**DOI:** 10.3390/cells15201906

**Published:** 2026-10-20

**Authors:** Jadier Colón-Vázquez, Yanitza Hernández-Santiago, Christian J. Malpica-Nieves, Nathaly M. Rosado-Rivera, Joshua J. Navedo-Jackson, Arelys A. Angueira-Laureano, Miguel P. Méndez-González, Yuriy Kucheryavykh, Misty J. Eaton, Serguei N. Skatchkov, David E. Rivera-Aponte

**Affiliations:** 1 Department of Biochemistry, Universidad Central del Caribe, Bayamon, PR 00956, USA; yanitza.hernandez@uccaribe.edu (Y.H.-S.); 124jnavedo@uccaribe.edu (J.J.N.-J.); miguel.mendez3@upr.edu (M.P.M.-G.); yuriy.kucheryavykh@uccaribe.edu (Y.K.); misty.eaton@uccaribe.edu (M.J.E.); serguei.skatchkov@uccaribe.edu (S.N.S.); 2 School of Chiropractic, Universidad Central del Caribe, Bayamon, PR 00956, USA; 3 Department of Physiology, Universidad Central del Caribe, Bayamon, PR 00956, USA; 4 Department of Biochemistry, University of Puerto Rico Medical Science, San Juan, PR 00921, USA; nathaly.rosado@upr.edu; 5 School of Medicine, University of Puerto Rico Medical Science, San Juan, PR 00921, USA; arelys.angueira@upr.edu; 6 Department of Natural Sciences, University of Puerto Rico, Aguadilla, PR 00603, USA; 7 Department of Sciences and Technology, Antillean Adventist University, Mayagüez, PR 00680, USA; 8 Department of Biology, University of Puerto Rico, Bayamon, PR 00959, USA; david.rivera11@upr.edu

**Keywords:** hyperglycemia, diabetes, DNA methylation, DNMT1, AMPK, SIRT-1, Kir4.1, astrocytes, primary cell culture, brain, diabetic db/db mice

## Abstract

**Highlights:**

**What are the main findings?**

- Hyperglycemia downregulates expression of polyamine-sensitive Kir4.1 channels in astrocytes via two pathways: epigenetic and metabolic.
- Epigenetically, hyperglycemia induces gene methylation by stimulating DNA methyltransferase (DNMT-1), leading to gene inactivation and, consequently, to suggested downregulation of Kir4.1 channel expression.
- Metabolically, hyperglycemia induces inactivation of the AMPK/SIRT-1 cascade, leading to downregulation of Kir4.1 channel expression in astrocytes.

**What are the implications of the main findings?**

- Since hyperglycemia engages dual regulatory pathways—(i) epigenetic via DNMT-1 and (ii) metabolic via AMPK/SIRT1—both DNMT-1 inhibition and AMPK activation are suggested as identified candidate pathways involved in the restoration of Kir4.1 expression under hyperglycemic conditions.
- Since SAM is involved not only in DNA methylation but also serves as an aminopropyl donor in polyamine synthesis, the polyamine strategy warrants further investigation because amino group-enriched drugs such as aminoguanidine and metformin restore Kir4.1 expression, as we showed previously.

**Abstract:**

The effects of chronic hyperglycemia on CNS, glial homeostasis and Kir4.1 regulation is poorly understood. Elevated blood glucose levels affect polyamine-sensitive inwardly rectifying potassium channels, Kir4.1, in astrocytes and retinal Müller glial cells. These cells regulate extracellular potassium and clear glutamate, processes supported by Kir4.1. Dysregulation of Kir4.1 channels has been implicated in glutamate toxicity, epilepsy, ataxia, and diabetes-induced neurodegeneration. In this study, we used Tandem Mass Tag Proteomic analysis, Pyrosequencing, SDS-PAGE and Western blot analysis, in whole brain and astrocytic primary cultures in hyperglycemic conditions testing DNMT1 and AMPK activities with RG-108, compound C, and AICAR treatments to investigate how hyperglycemia alters Kir4.1 expression in astrocytes and explored two regulatory mechanisms: (i) DNA methylation mediated by DNA methyltransferase 1 (DNMT-1), which uses S-adenosylmethionine (SAM) as a methyl donor, and (ii) metabolic signaling via the AMPK/SIRT-1 cascade. We found that hyperglycemia (25 mM glucose) (i) increased DNMT-1 protein levels in astrocytes by 1.5–2-fold and (ii) decreased AMPK and (iii) SIRT-1 levels, accompanied by a reduction in Kir4.1 protein expression. Pharmacological inhibition of DNMT-1 with RG-108 decreased DNMT-1 levels and restored Kir4.1. Treatment with AICAR, a selective AMPK activator, rescued SIRT-1 and Kir4.1 expression. In summary, hyperglycemia engages dual regulatory pathways: (i) epigenetic via DNMT-1 and (ii) metabolic via AMPK/SIRT-1, which converge on Kir4.1 downregulation in astrocytes. Therefore, both DNMT-1 inhibition and AMPK activation rescued Kir4.1 expression, suggesting functional support for potassium and glutamate homeostasis and membrane potential stability in glia.

## 1. Introduction

Diabetes is characterized by elevated blood glucose levels that cause hyperglycemia and disrupt normal central (CNS) and peripheral (PNS) nervous system function, particularly by affecting polyamine (PA)-sensitive inwardly rectifying Kir4.1 potassium channels in hyperglycemic astrocytes [1,2,3], in retinal Müller glial cells in chemically induced diabetes in rats [4], and in diabetic db/db mice [5,6,7]. Diabetes mellitus is a metabolic disorder marked by chronic elevations in blood and brain glucose, which can dysregulate glial cell function [4,7,8]. Patients with diabetes face a heightened risk of seizures, stroke, and neurodegenerative conditions, yet mechanistic understanding remains limited [3,9]. In both astrocytic cell culture [10] and animal brain slices, reduced Kir4.1 channel expression and function altered astrocyte membrane potentials and impaired K^+^ and glutamate uptakes [6,7,11]. Furthermore, db/db mice with type 2 diabetes demonstrate reduced Kir4.1 channel expression, which is associated with epileptogenic hyperexcitability in the hippocampus [6], most probably due to impaired glutamate uptake observed when Kir4.1 channels were knocked down [10]. Moreover, hyperglycemia has been reported to modulate the small non-coding RNAs (e.g., miR-205) that downregulate Kir4.1 [2].

Mutations in inwardly rectifying potassium channels, particularly Kir4.1, also lead to epilepsy, ataxia, hypokalemia, hypomagnesemia, and deafness in EAST-SeSAME syndrome [12,13,14]. Dysregulation of Kir4.1 has been linked to reduced glutamate uptake [10], which is implicated in glutamate toxicity and diabetes-induced neurodegeneration [15,16]. In the db/db mouse model of diabetes, astrocytic Kir4.1 channel expression is markedly reduced [7], mirroring findings in hyperglycemic rat astrocytes [3]. However, amino group-enriched drugs such as aminoguanidine and metformin (non-classical polyamines) restore Kir4.1 protein levels under diabetic conditions [3,5].

Kir4.1 channel currents are regulated by polyamines (PAs), which bind DNA, RNA, and ATP [17] and aggregate to protect DNA structure [18], playing a role in DNA methylation. PAs rectify Kir4.1 by blocking outward potassium currents through the channel [19,20,21]. This rectification is a key requirement for normal Kir4.1 function [14,21]. Mutations that abolish PA-dependent Kir4.1 channel rectification have been observed in many diseases and syndromes [14,21,22,23]. Intriguingly, endogenous PAs such as spermidine (SPD) and spermine (SPM) accumulate in astrocytes, not neurons [24,25,26,27], together with Kir4.1 channels [28] and Cx43 channels [29,30,31], allowing large negatively charged molecules to pass through GJs in astrocytic syncytium, and to promote molecular signaling between astrocytes [32,33].

**Scheme 1:**
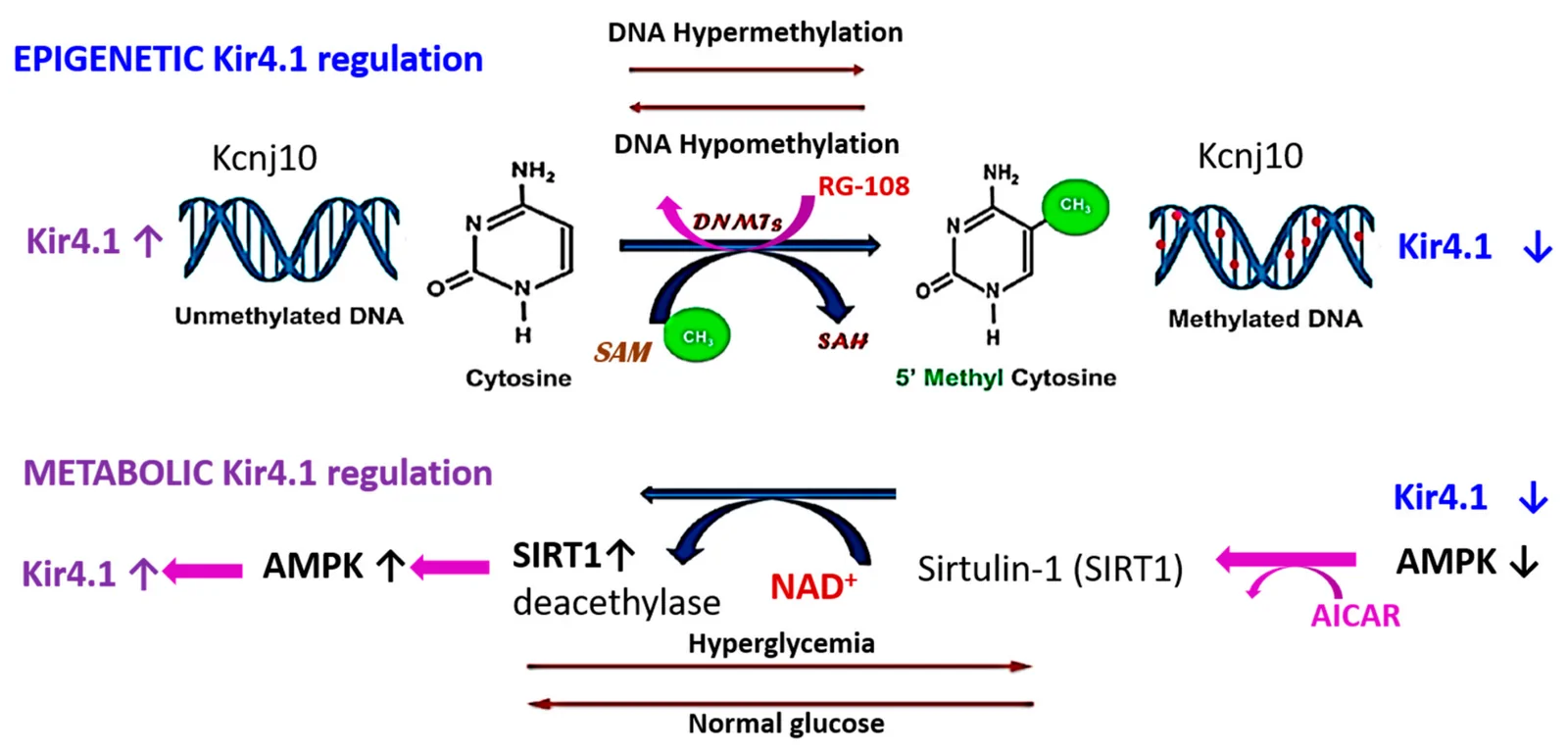
Hypothesis: Hyperglycemia differentially regulates Kir4.1 channel expression in astrocytes. We hypothesize that hyperglycemia results in (i) removing an inhibitory control over DNA methyltransferase 1 (DNMT-1) due to suppression of AMP-activated protein kinase (AMPK), thereby (ii) allowing aberrant methylation of the Kir4.1 (Kcnj10) locus, while simultaneously reducing Sirtuin-1 (SIRT-1) deacetylase activity. However, (iii) depression of DNMT1 (by RG-108) or (iv) activation of the AMPK-SIRT1 cascade (by AICAR) will restore Kir4.1 expression. These combined epigenetic and metabolic pathways regulate Kir4.1 expression and astrocyte homeostasis. We suggest that both epigenetic and metabolic pathways converge on Kir4.1 regulation. DNMT-1 represses Kir4.1 gene (Kcnj10) expression, while AMPK and its downstream effector, SIRT-1, act as metabolic regulators of channel expression.

The Kcnj10 gene, which encodes the Kir4.1 protein, must be activated for Kir4.1 channel subunit production; this is why DNA methylation is a key regulator of Kir4.1 protein synthesis during astrocyte maturation [34]. Hypermethylation of the Kcnj10 gene has been observed in models of brain trauma, where Kir4.1 is downregulated, whereas hypomethylation may restore Kir4.1 (Scheme 1) [35]. For DNA methylation, S-adenosylmethionine (SAM) serves as the primary methyl-group donor for DNA methyltransferases (DNMTs), thereby influencing gene-silencing enzyme activities (Scheme 1). Astrocyte-specific genes undergo dynamic methylation changes during development, but knockout of DNMT-1 in neural precursor cells causes hypomethylation and premature astrocyte differentiation [35]. In summary, SAM activity is linked to DNMT activity and impacts Kir4.1 synthesis in trauma, aging, and disease. Epigenetic modifications, especially DNA methylation mediated by DNMT-1, regulate gene expression without altering DNA [34,35].

Conversely, regarding the metabolic regulation of Kir4.1 expression, an adenylate charge-regulated protein kinase, also called 5′-adenosine monophosphate (AMP)-activated protein kinase (AMPK), is a master cellular enzyme that senses low energy and regulates nutrient metabolism in response to cellular energy status. In low-energy states, AMPK is activated by phosphorylation and upstream kinases, increasing glucose uptake [36]. AMPK regulates inflammation, often via the Sirtuin-1 protein (SIRT-1), a NAD^+^-dependent deacetylase that suppresses pro-inflammatory transcription by deacetylating transcriptional regulators, including nuclear factor kappa-B (NF-κB) [37]. Under hyperglycemic conditions, AMPK activity is often suppressed, thereby exacerbating metabolic stress [38].

Chronic hyperglycemia often triggers low-grade inflammation in the CNS, elevating pro-inflammatory cytokines that contribute to neuronal injury and increase stroke risk [39]. SIRT-1 is a critical enzyme that uses cellular energy (via NAD^+^) to remove acetyl groups from other proteins, acting as a key metabolic sensor and stress-response regulator. SIRT1 regulates this response by suppressing death and inflammatory signals such as p53 and NF-κB, promoting DNA repair, and reducing oxidative stress [37,40]. AMPK has been shown to activate SIRT-1 (for example, by increasing cellular NAD^+^), and SIRT-1 deacetylates upstream activators such as liver kinase-1 (LKB1), a tumor-suppressing serine/threonine kinase (STK11), feeding back into the AMPK pathway and forming a regulatory loop [36]. Our hypothesis is that hyperglycemia suppresses AMP-activated protein kinase (AMPK), thereby removing an inhibitory control over DNA methyltransferase 1 (DNMT-1) and allowing aberrant methylation of the Kir4.1 (Kcnj10) locus, while simultaneously reducing Sirtuin-1 (SIRT-1) deacetylase activity. These combined epigenetic and metabolic disruptions result in reduced Kir4.1 expression and impaired astrocyte homeostasis. We suggest that both epigenetic and metabolic pathways converge on Kir4.1 regulation. DNMT-1 represses Kir4.1 gene (Kcnj10) expression, while AMPK and its downstream effector, SIRT-1, act as metabolic regulators of channel expression and inflammation. Despite this, the precise molecular mechanisms by which hyperglycemia disrupts astrocytic homeostasis remain unknown.

## 2. Materials and Methods

### 2.1. Animals

For our Tandem Mass Tag Proteomics, we utilized both male and female mice: homozygous (db/db) diabetic mice and heterozygous (db/+) control mice. We obtained our animals from Jackson Laboratories (Bar Harbor, ME, USA). We kept the animals in a 12 h light and dark cycle, and their access to food and water was not restricted until glucose testing. The Institutional Animal Care and Use Committee (IACUC) at the Universidad Central del Caribe approved all experiments conducted in this study. Db/db diabetic and db/+ control mice were used for proteomic analysis (protocol #051-2023-31-91-PHA), and Sprague Dawley rats were used for astrocyte cultures to study hyperglycemia (protocol #058-2023-13-000), following the National Institutes of Health guidelines for the humane treatment of laboratory animals.

The data presented in this manuscript were generated specifically for this manuscript; no results, figures, or raw data from previously published studies were used. References to prior publications are provided solely to contextualize the findings and highlight consistency across independent experiments, not to indicate reuse of previously published datasets.

### 2.2. Tandem Mass Tag Proteomics

We used whole-brain extraction from eight-week-old male leptin receptor-deficient diabetic mice (db/db) and age-matched heterozygous controls (db/+) as the experimental model. Animals were housed under standard conditions with ad libitum access to food and water. At 8 weeks of age, mice were euthanized, and brain tissue was rapidly dissected on ice, snap-frozen in liquid nitrogen, and stored at −80 °C until processing. Brain tissue was homogenized in ice-cold lysis buffer containing protease and phosphatase inhibitors. Samples were analyzed by LC-MS/MS using a nano-LC system (UltiMate 3000, LC Packings, Dionex Product, ThermoScientific, Waltham, MA, USA) coupled to an LTQ-Orbitrap XL mass spectrometer (Thermo, San Jose, CA, USA). Peptides were loaded onto a nano-precolumn (PepMapTM C18, 5 μm, 100 Å, 300 µm I.D. × 1 mm, 5 pcs; LC Packings) and eluted in line with an analytical column (C18, 2.6 μm, 35 cm, ref. MPL-ES-2.6C18, Teknokroma, Barcelona, Spain) at 430 nL/min and using a continuous acetonitrile gradient consisting of 0–12% B for 1 min, 12–45% B for 120 min, 45–90% B for 2 min, 90% B for 7 min (solvent A: 0.1% (*v*/*v*) formic acid; solvent B: 90% (*v*/*v*) acetonitrile, 0.1% (*v*/*v*) formic acid). LTQ-Orbitrap XL was operated in a data-dependent mode with a normal FT-resolution spectrum (resolution = 100,000) followed by the MS/MS spectra from the most intense ten parent ions, using CID normalized collision energy (35 eV), 2 microscans, and 90 s dynamic exclusion time. Protein identification was performed using the Proteome Discover 1.4.0.288 package (Thermo), allowing optional (methionine oxidation, lysine and arginine modification of +4 Da) and fixed modifications (cysteine carboxamidomethylation), 2 missed cleavages, 20 ppm tolerance for the precursor and 1.2 atomic mass units mass tolerance for fragment ions. MS/MS raw files were searched against the Uniprot Rat data base. Proteins were identified using 1% FDR.

### 2.3. Pyrosequencing

Genomic DNA was extracted from mouse brain tissue samples using either a standard phenol-chloroform method or a commercial column-based DNA extraction kit, following the manufacturer’s instructions (Cat#D3024, Zymo Research, Irvine, CA, USA). DNA concentration and purity were assessed using NanoDrop. The genomic DNA was obtained from astrocytes that were cultured under normal-glucose (5 mM) or high-glucose (25 mM) conditions. DNA methylation profiling was carried out by Zymo Research (Irvine, CA, USA) using whole-genome bisulfite sequencing (WGBS). A total of six biological samples were examined, including three independent biological replicates from the 5 mM glucose group and three independent biological replicates from the 25 mM glucose group. The genomic DNA was treated with bisulfite and prepared for whole-genome bisulfite sequencing according to the manufacturer’s validated WGBS procedure. The bisulfite-converted DNA libraries were then amplified, given an index, and subjected to high-throughput sequencing on an Illumina sequencing platform. The sequencing reads were quality-filtered and aligned to the relevant reference genome, and the methylation status was determined at the single-base level by identifying methylated cytosines as opposed to the bisulfite-converted unmethylated cytosines. Genome-wide DNA methylation profiles were produced for each biological replicate. Differentially methylated cytosites (DMCs) between the 5 mM and 25 mM glucose groups and differentially methylated regions (DMRs) were found using the bioinformatic pipeline supplied by Zymo Research.

### 2.4. Astrocyte Primary Cultures for Hyperglycemic Research

Our primary astrocyte culture utilized the neocortex of newborn Sprague Dawley rats, aged 1–3 days old, in accordance with the protocol [3,11] approved by the Institutional Animal Care and Use Committee (protocol #058-2023-13-000). Briefly, after decapitation of the animals, the brains were stripped of their meninges to minimize the contamination of the culture with fibroblasts. Using the stomacher blender methods, we disassociated the forebrain cortex, and cell suspension was filtered via gravity, first through a #60 sieve and then with a #100 sieve. Suspension was centrifuged and divided into two groups, each in their respective uncoated 75 cm^2^ flasks at a density of 300,000 cells/cm^2^. Our treatment group was plated in high-glucose Dulbecco’s Modified Eagle Medium (DMEM) containing 25 mM glucose, 2 mM glutamine, 1 mM pyruvate, 10% fetal bovine serum, and 100 iU/mL penicillin/100 μg/mL streptomycin. Our control group was plated in normal-glucose DMEM, with a glucose concentration of 5 mM. The medium was replaced with the appropriate fresh culture medium every 3 days. In the second week of culture, the mixed glial cultures were treated with 50 mM L-Leucine methyl ester (LME) (pH 7.4) for 45 min to eliminate microglia. The recovery period before treatment or experimentation was at least 1 day. Astrocytes were dissociated by trypsinization and reseeded onto appropriate plates for the experiments.

### 2.5. SDS-PAGE and Western Blot Analysis

After treatment of the cells, we scraped the cells from their respective plates and proceeded to perform a protein extraction. We utilized a lysis buffer containing phenylmethylsulfonyl fluoride (PMSF), protease inhibitor, and Radioimmunoprecipitation Assay (RIPA) buffer, the latter of which contained Tris-HCl 1.5 M, pH 8.8, 1% Triton X-100, 150 mM NaCl, and 0.1% SDS. Once lysed, we incorporated Urea sample buffer with β-Mercaptoethanol. We boiled and centrifuged briefly to pellet the debris. For the majority of samples, they were immediately run on 10% SDS-polyacrylamide gels, and leftover samples without the Urea sample buffer were stored at −80 °C. The protein concentration of cell homogenates was determined via a Bradford assay (Bio-Rad, Hercules, CA, USA); afterwards, an appropriate volume of Urea sample buffer was added to our mix (62 mM Tris/HCl pH 6.8, 4% SDS, 8 M Urea, 20 mM EDTA, 5% β-Mercaptoethanol, 0.015% Bromophenol Blue) to a final concentration of 15 μg protein/μL, and incubation in a water bath at 100 °C for 10 min took place.

### 2.6. Western Blotting

Following a Western blot protocol previously described [3], we prepared our antibodies in 5% Bovine Serum Albumin (BSA). Our specific antibodies were Kir4.1 (1:2000, Cat #: APC-035-GP Alomone, Jerusalem, Israel), DNMT-1 (1:1000, Cat # PA1880, Invitrogen, Waltham, MA, USA), and SIRT-1 (1:1000, Cat # 19A7AB4 Abcam, Cambridge, UK). We utilized enhanced chemiluminescence methodology (SuperSignal^®^ West Dura Extended Duration Substrate; Pierce, Rockford, IL, USA), as described by the manufacturer, for our final detection. The signal intensity was measured using a gel documentation system (Versa Doc Model 1000, Bio-Rad, Hercules, CA, USA). For all experiments utilizing Western blot, the chemiluminescence intensity was corrected to account for minor differences in protein content after India ink-stained densitometric analysis of the membranes.

### 2.7. RG-108 Treatment

Cells were treated with the DNA methyltransferase inhibitor RG-108 (Cat # 48208-26-0, MedChemExpress. Monmouth Junction, NJ, USA) at 100 µM in DMEM-free media for 4 days. After treatment, cells were collected and lysed for protein analysis.

### 2.8. AICAR Treatment

Cells were treated with AICAR (5-aminoimidazole-4-carboxamide ribonucleoside), a specific activator of AMPK (Cat # 2627-69-2 MedChemExpress. Monmouth Junction, NJ, USA) at a concentration of 100 μM, diluted in DMEM-free media for 24 h. After treatment, cells were collected and lysed for protein analysis.

### 2.9. Compound C Inhibitor Treatment

Cells were treated with compound C at a concentration of 100 µM, diluted in DMSO for 24 h (Cat # 866405-64-3, MedChemExpress. Monmouth Junction, NJ, USA). After treatment, cells were collected and lysed for protein analysis.

### 2.10. DNMT-1 Activity Assay

#### 2.10.1. Reagent Preparation

DNMT-1 enzymatic activity was quantified using a commercially available colorimetric assay kit (Cat # ab113467, Abcam, Cambridge, UK) following the manufacturer’s instructions. A 1× wash buffer was prepared by diluting 13 mL of 10× wash buffer with 117 mL of distilled water and adjusting the pH to 7.2–7.5; the solution was stored at 4 °C for up to six months. The Adomet working buffer was freshly prepared before use by adding 2 µL of 50× Adomet to 98 µL of DNMT assay buffer. Capture antibody, detection antibody, and enhancer solutions were diluted in 1× wash buffer at 1:1000, 1:2000, and 1:5000, respectively. Nuclear extracts containing DNMT activity served as experimental samples.

#### 2.10.2. Activity Assay Protocol

The required number of strip wells was predetermined, and all samples were analyzed in replicate, including blank and positive control wells. Blank wells received 50 µL of Adomet working buffer, while positive control wells received 50 µL of Adomet working buffer supplemented with 1 µL of DNMT enzyme control. Experimental wells received 45–49 µL of Adomet working buffer with 1–5 µL of nuclear extract or purified DNMT enzyme, adjusted to a final volume of 50 µL. For inhibitor-treated conditions, 5 µL of inhibitor solution was included, and the buffer volume was adjusted accordingly. Plates were sealed and incubated at 37 °C for 90–120 min. Following incubation, wells were aspirated and washed three times with 150 µL of 1× wash buffer. Subsequently, 50 µL of diluted capture antibody was added to each well and incubated for 60 min at room temperature. Wells were washed three times before adding 50 µL of diluted detection antibody and incubating for 30 min, followed by four washes. Next, 50 µL of diluted enhancer solution was added and incubated for 30 min at room temperature, followed by five washes. Color development was initiated with 100 µL of developer solution and monitored for 1–10 min in the absence of direct light. The reaction was terminated by adding 100 µL of stop solution when the positive control wells reached a medium blue color. Absorbance was measured within 2–10 min at 450 nm using a microplate reader, with 655 nm as an optional reference wavelength.

### 2.11. Statistical Analysis

TMT reporter ion intensities were extracted from MS/MS spectra and used to quantify relative protein abundance across experimental groups. Data were normalized across all TMT channels to correct for loading and labeling variability, and protein-level ratios were derived from aggregated peptide intensities. Statistical significance of differential protein expression (e.g., db/db vs. control) was assessed using *t*-tests or ANOVA, followed by false discovery rate (FDR) correction to account for multiple comparisons.

Pyrosequencing data were analyzed using dedicated methylation quantification software, and peak heights from the programs were used to determine the relative incorporation of cytosine versus thymine at each *CpG* site. Methylation percentages were calculated as *C*/*(C* + *T*) signal ratios and averaged across technical replicates for each condition (5 mM vs. 25 mM glucose). Statistical significance between groups was assessed using Student’s *t*-test or ANOVA, and the results were reported as mean ± SEM, with significance set at *p* < 0.05.

Western blot signal analysis, quantification and normalization were performed using Image Lab (Bio-Rad, Hercules, CA, USA). Western blot quantification for groups of treatment vs. control was analyzed using a Student’s *t*-test, and experiments with multiple groups (one control group and three treatment groups) were analyzed with one-way ANOVA followed by Tukey’s multiple-comparison test. A value of *p* < 0.05 was considered significant. The collected Western blot data files were analyzed using GraphPad Prism v10.4.1 (GraphPad, San Diego, CA, USA).

## 3. Results

### 3.1. TMT Proteomics Shows That Diabetic Mice Have a Key Astrocytic Protein Downregulated Compared with Controls

**Figure 1:**
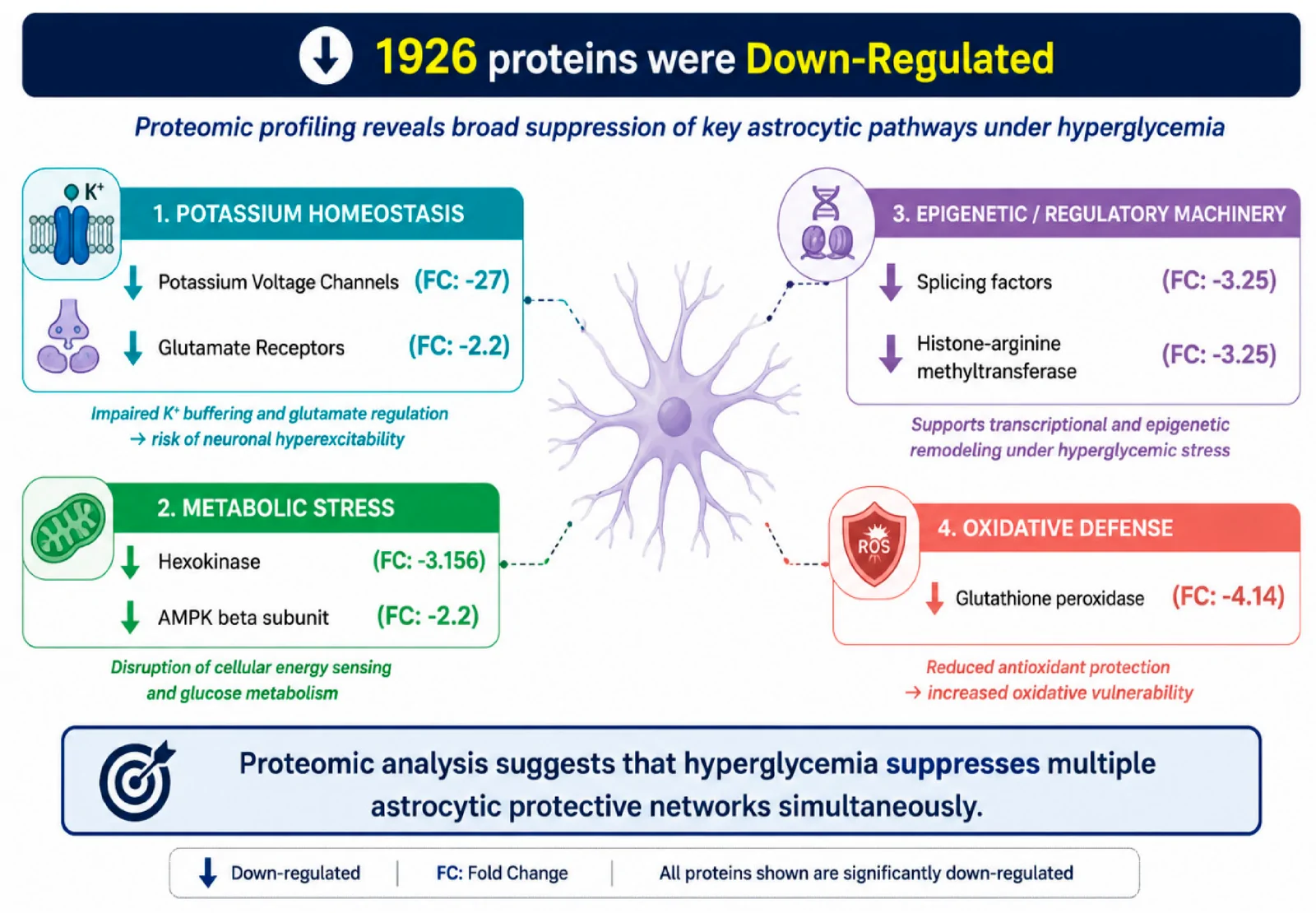
Downregulation of key proteins in the db/db mice brain under hyperglycemic conditions; proteomic analysis. Eight-week-old db/db mice were dissected, and the whole brain was utilized and processed for proteomics. 1926 proteins were downregulated; key proteins involved in potassium homeostasis, metabolic regulation, oxidative defense, and epigenetics were downregulated. Note: Potassium channels were downregulated most significantly in db/db mouse brains (down 27-fold).

Using whole-brain extraction from 8-week-old control mice and diabetic db/db mouse models, we extracted the brains and performed Tandem Mass Tag Proteomics. Our results showed that 308 proteins were upregulated in diabetic mice vs. controls. Among these proteins, histones, Glial Maturation Factor, and glyceraldehyde-3-phosphate dehydrogenase stood out (Figure 1). Conversely, we identified 1926 proteins downregulated in diabetic mice compared with controls (Figure 1). Within these proteins, we found that possible astrocytic pathways are downregulated in diabetic mice; proteins belonging to potassium homeostasis, metabolic stress, epigenetic regulation, and oxidative stress are downregulated from 2- to 4-fold, while potassium channel proteins were downregulated most significantly, by 27-fold. These results suggest that hyperglycemic conditions widely affect the astrocytes and their metabolic function, which can lead to the degeneration of neurons.

### 3.2. Primary Astrocytes from Rat Cortex Grown in High-Glucose Conditions Have Higher Methylation on the CpG Island of Key Genes Related to Astrocyte Function

**Figure 2:**
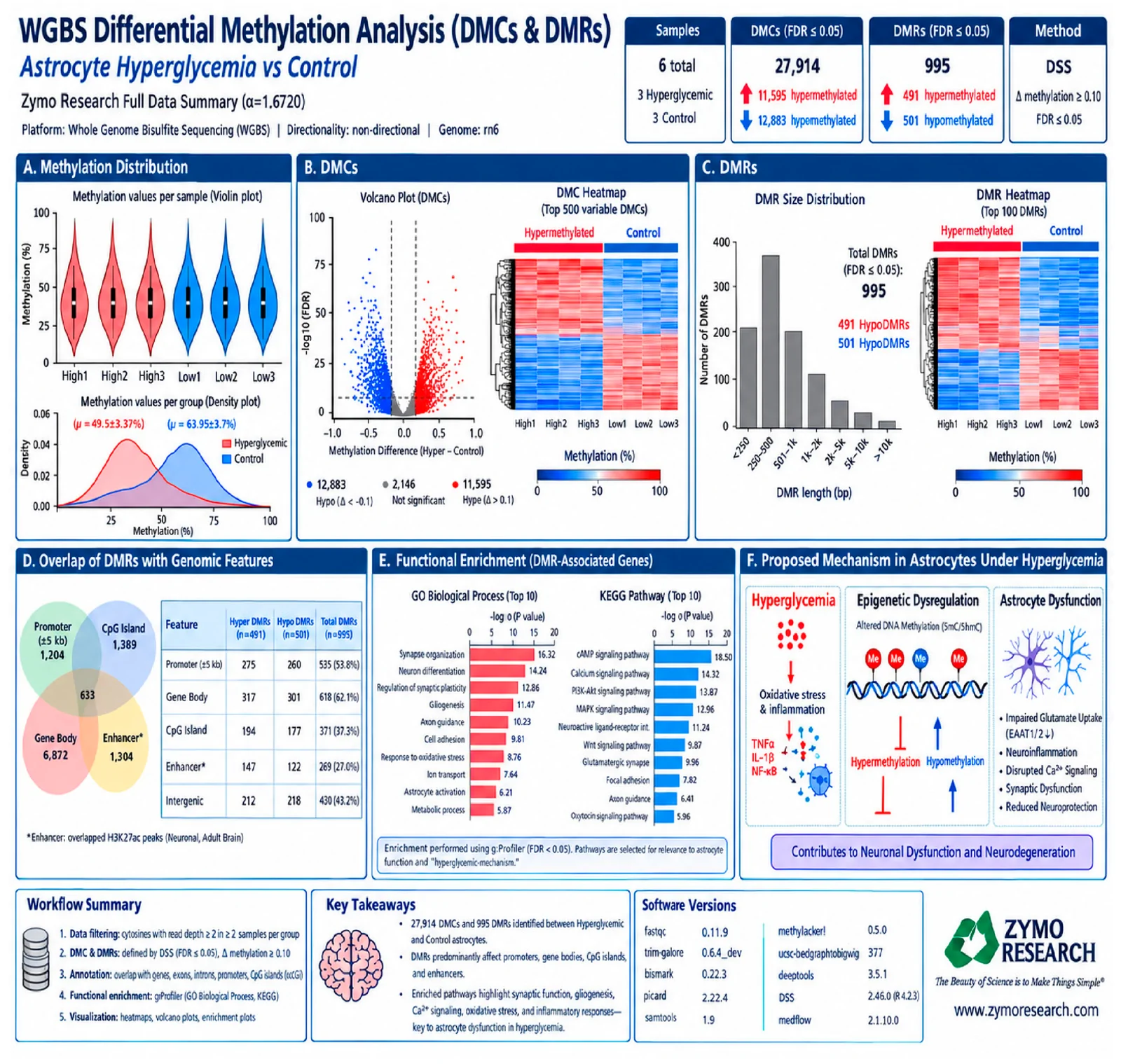
Hyperglycemia induces a large-scale and organized epigenetic response, characterized by both localized *CpG* remodeling and coordinated regional methylation changes. Astrocytes were maintained in culture for two weeks under either normal (5 mM) or high (25 mM) glucose conditions, followed by gDNA extraction for pyrosequencing. Hyperglycemia induced extensive genome-wide epigenetic remodeling, with 27,914 significantly differentially methylated cytosines (DMCs) identified, of which 24,478 passed adjusted significance criteria, including 12,883 hypomethylated and 11,595 hypermethylated loci. Differentially methylated region (DMR) analysis identified 995 coordinated methylation regions, comprising 491 hypomethylated and 501 hypermethylated regions, indicating that methylation changes occurred in organized genomic clusters rather than isolated *CpG* events. The balanced distribution of hypo- and hypermethylation supports a model of selective, large-scale epigenetic remodeling in response to hyperglycemic exposure, with potential implications for transcriptional regulation.

A Cytosine-phosphate-Guanine (*CpG*) island, where a cytosine (C) is linked to a guanine (G) by a phosphate group in the 5′ → 3′ direction, is a key “island” for DNA methylation. Earlier reports show that DNA methylation on the *CpG* island of diabetic mice can target the Kcnj10 gene in spinal cord injury [41], yet it is unknown whether it also targets the Kcnj10 gene in isolated astrocytes. We used primary rat astrocytes cultured in normal-glucose (5 mM) and high-glucose conditions (25 mM). After a 2-week growth period, we extracted genomic DNA (gDNA), a complete set of chromosomal genetic material from astrocytes, and evaluated it via pyrosequencing to determine whether the *CpG* islands are over-methylated in astrocytes grown in high-glucose conditions compared with controls (Figure 2).

Hyperglycemia induces widespread, bidirectional epigenetic remodeling characterized by differential methylation at both single-*CpG* and regional levels (Figure 2). Genome-wide DNA methylation analysis identified 27,914 significantly differentially methylated cytosines (DMCs) across experimental conditions, of which 24,478 loci passed the adjusted significance threshold and were retained for downstream analysis. Among these significant DMCs, 12,883 exhibited hypomethylation, whereas 11,595 demonstrated hypermethylation, indicating that hyperglycemic exposure promotes extensive, directionally balanced alterations in the methylome rather than a globally uniform methylation shift (Figure 2). The presence of both methylation gain and loss events suggests selective epigenetic remodeling, consistent with targeted regulation of genomic regions associated with cellular adaptation to hyperglycemic stress.

At the regional level, analysis identified 995 differentially methylated regions (DMRs), including 491 hypomethylated and 501 hypermethylated regions, demonstrating coordinated methylation changes across neighboring Cytosine-phosphate-Guanine (*CpG*) sites. Because DMRs represent clusters of concordant methylation alterations rather than isolated *CpG* events, these regions are generally considered to possess greater biological relevance and regulatory potential. Regional methylation changes are more strongly associated with modulation of chromatin state and transcriptional activity and are less susceptible to stochastic variability than individual DMC events (Figure 2). Collectively, these findings demonstrate that hyperglycemia induces a large-scale and organized epigenetic response, characterized by both localized *CpG* remodeling and coordinated regional methylation changes that may contribute to downstream transcriptional regulation. The results demonstrate that key genes in the metabolic, inflammatory, and epigenetic regulatory pathways are hypermethylated, which suppresses their expression, resulting in reduced protein expression and possibly causing a disruption in astrocyte functions. Conversely, astrocytes grown in high-glucose conditions show decreased methylation of oxidative stress genes, which are activated once ROS levels are elevated and the astrocytes respond to stress (Figure 2).

### 3.3. Astrocytes Grown in High-Glucose Conditions Show an Increase in the Protein Expression of DNMT-1

**Figure 3:**
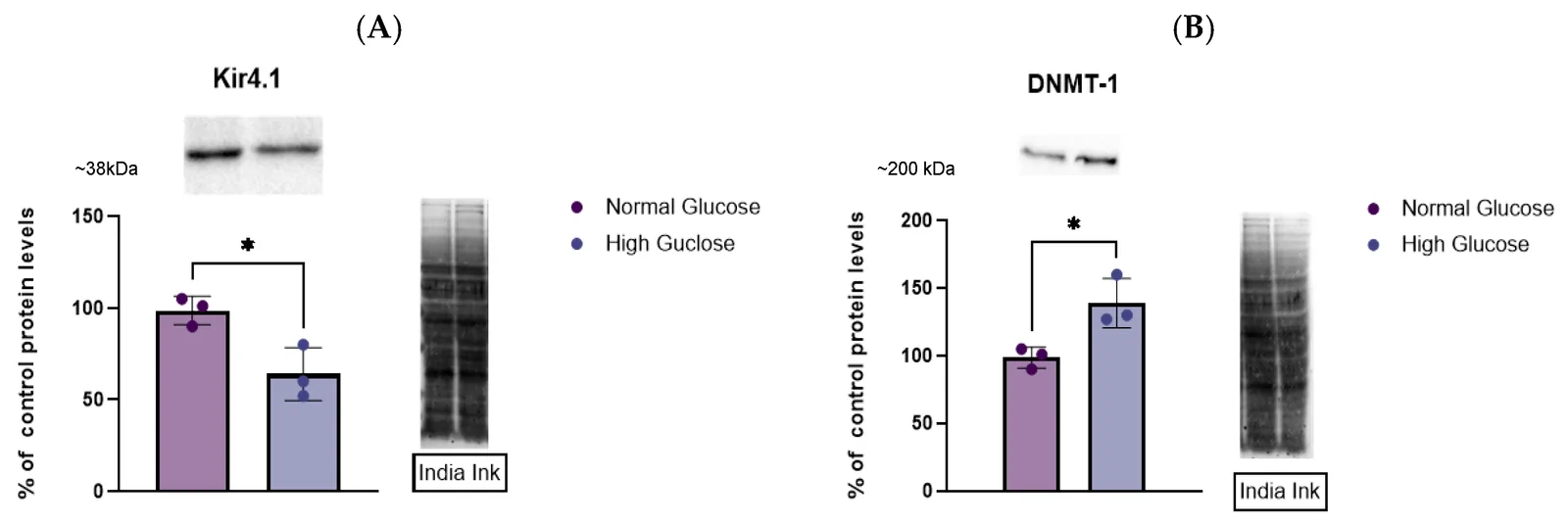
Kir4.1 protein level is decreased, but DNMT-1 level is increased, under hyperglycemic conditions. Astrocytes were maintained under normal (5 mM) or high (25 mM) glucose conditions for two weeks, following protein extraction and analysis by Western blotting. (**A**) Kir4.1 protein expression was significantly reduced in astrocytes exposed to hyperglycemic conditions relative to control cells (34.67% of control ± 9.4 SEM, n = 3). (**B**) DNMT-1 protein expression was significantly elevated in astrocytes maintained under hyperglycemic conditions compared with controls. (140.33% of control ± 11.45 SEM, n = 3). Quantification was normalized utilizing India ink (* *p* < 0.05).

Following our pyrosequencing experiment, we hypothesized that high glucose elevates DNMT-1 expression. To test this hypothesis, we cultured astrocytes in normal-glucose (5 mM) and high-glucose (25 mM) conditions. After a two-week growth period, we treated them with LME to eliminate microglia and ensure a pure culture of astrocytes. After treatment, cells were collected and lysed for protein extraction. Protein quantification was performed with the Bradford assay, and protein levels were determined via Western blot. As illustrated in Figure 3A, astrocytes maintained under hyperglycemic conditions exhibited a significant reduction in Kir4.1 protein expression compared with control cells (34.67% of control ± 9.4 SEM, *n* = 3).

As shown in Figure 3B, astrocytes maintained under hyperglycemic conditions exhibited a significant increase in DNMT-1 protein expression relative to control cells (140.33% of control ± 11.45 SEM, n = 3). India ink staining was used for normalization of the quantitative measurements (*p* < 0.05). Collectively, our findings demonstrate that astrocytes maintained under high-glucose conditions exhibit reduced Kir4.1 protein expression (Figure 3A) and elevated DNMT-1 protein expression (Figure 3B) relative to those cultured under normal-glucose conditions.

### 3.4. DNMT-1 Has Elevated Activity Levels in Astrocytes Grown in High-Glucose Conditions When Compared to Controls

Taken together, our findings indicate increased methylation and increased protein expression in astrocytes maintained under high-glucose conditions. Based on these observations, we hypothesized that DNMT-1 activity is elevated in astrocytes exposed to high-glucose conditions relative to control cells.

**Figure 4:**
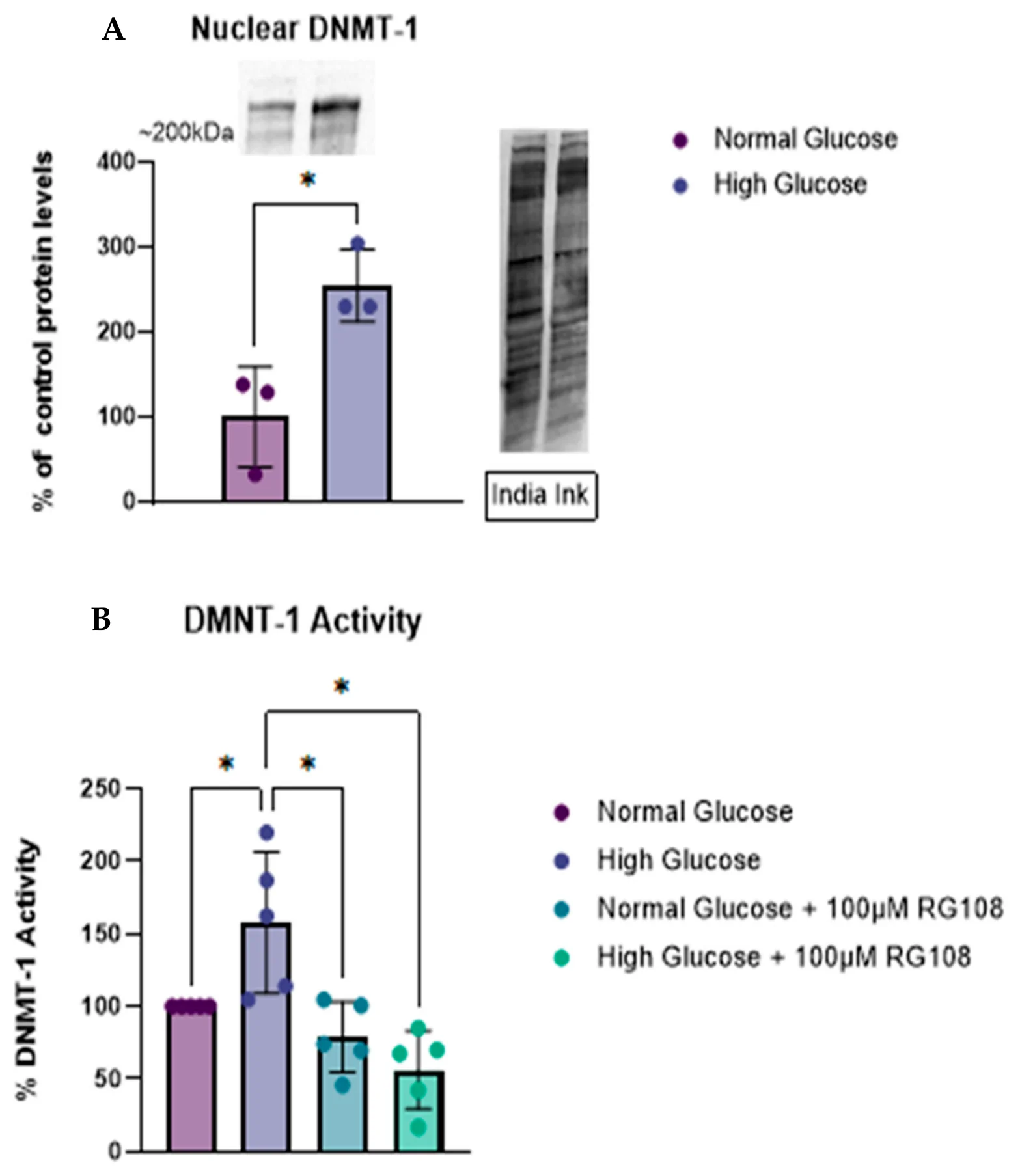
Nuclear DNMT-1 levels and DNMT-1 activity with and without RG 108. Astrocytes were cultured in normal (5 mM) and high (25 mM) glucose conditions for two weeks followed by treatment with RG-108 at 100 µM for 4 days. Nuclear protein extraction for protein measurements was via Western blot. (**A**). Nuclear DNMT-1 protein expression was significantly increased in astrocytes cultured in hyperglycemic conditions when compared to controls (154.7% of control ± 41.88 SEM, n = 3). (**B**). Nuclear DNMT-1 activity was significantly increased between normal glucose and high glucose without treatment (*p* = 0.037, 95% C.I. = [−112.3 to −2.767]). There was also a significant difference between high glucose and high glucose with RG-108 (*p* = 0.0003, 95% C.I. = [46.33 to 155.8], n = 5). Quantification was normalized utilizing India ink (* *p* < 0.05).

To further evaluate the effects of DNMT-1 in astrocytes, we performed primary astrocyte cultures using control DMEM with 5 mM glucose and high-glucose DMEM with 25 mM. Following two weeks of culture, cells were seeded into six-well plates at a density of 300,000 cells per well and subsequently incubated with 100 µM RG-108 (N-phthalyl-L-tryptophan), a DNMT inhibitor, for four days. We then performed a nuclear extraction to evaluate DNMT-1 protein levels and measure activity in the nuclear extract. Our findings demonstrate that DNMT-1 protein expression, assessed in whole-cell lysates, is significantly elevated in astrocytes maintained under hyperglycemic conditions relative to control cells (154.7% of control ± 41.88 SEM, n = 3). Quantification was normalized using India ink (* *p* < 0.05) (Figure 4A). DNMT-1 activity is markedly elevated in astrocytes maintained under high-glucose conditions compared to control cells (Figure 4B). However, when treated with the selective inhibitor RG-108, activity decreased compared with untreated astrocytes (Figure 4B).

### 3.5. Astrocytes Cultured in High-Glucose Conditions Have an Increased Kir4.1 Protein Expression When Treated with RG-108

**Figure 5:**
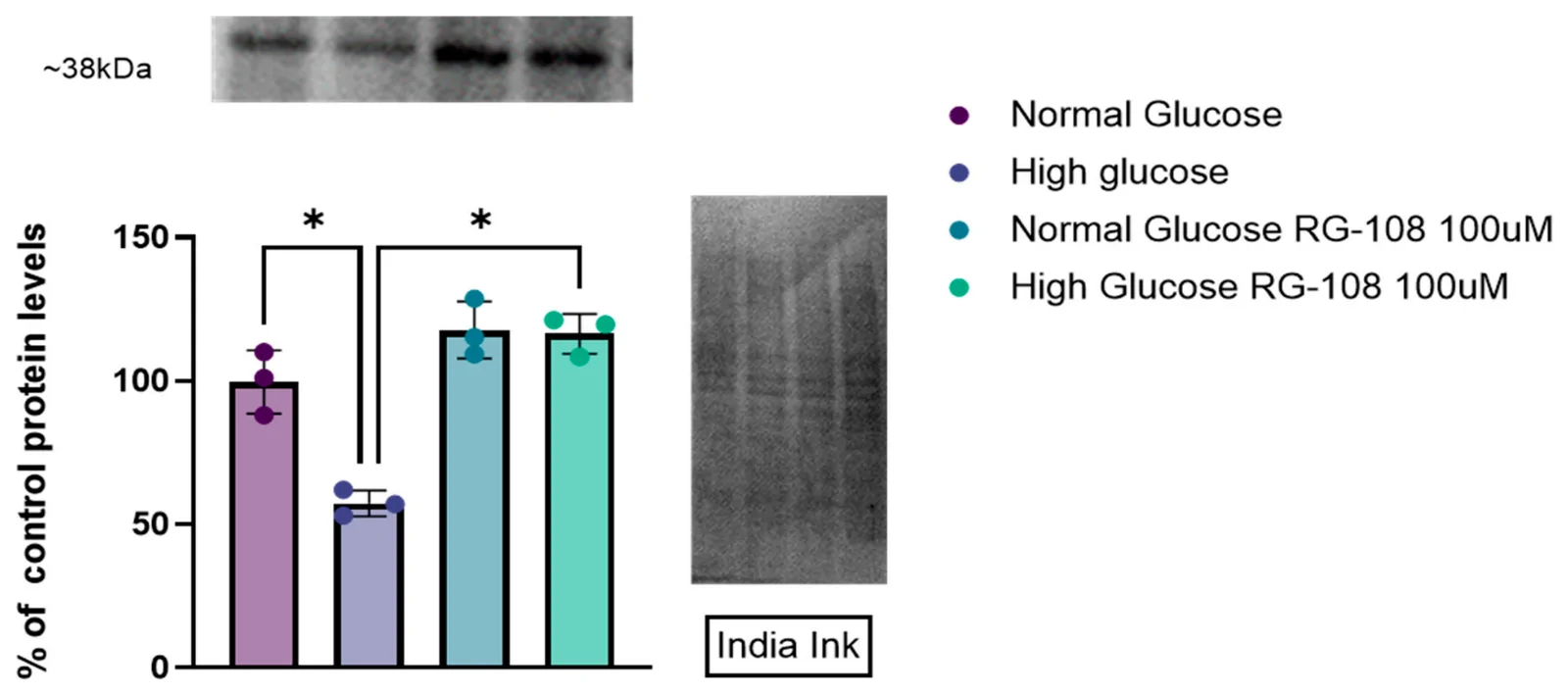
The Kir4.1 channel protein was significantly increased after RG 108 treatment in hyperglycemic conditions. Astrocytes were cultured in normal (5 mM) and high (25 mM) glucose conditions for two weeks, followed by treatment with RG-108 at 100 µM for 4 days. Protein extraction for protein measurements was via Western blot. Kir4.1 protein expression was significantly increased in astrocytes cultured in hyperglycemic conditions after treatment with RG-108 when compared to hyperglycemic astrocytes without treatment (59.06% of control ± 6.94 SEM, n = 3). Quantification was normalized utilizing India ink (* *p* < 0.05).

It has been shown that DNMT-1 bi-directionally regulates KCNJ10 expression and Kir4.1 protein expression [34]. Here, we show that hyperglycemia increased DNMT-1 expression, and RG-108 (N-phthaloyl-L-tryptophan), known as DNMT inhibitor, reduces expression in astrocytes maintained under high-glucose conditions compared to control cells (Figure 4). We hypothesized that treatment with RG-108 would increase the Kir4.1 protein expression in astrocytes. Astrocytes were cultured under normal (5 mM) or high (25 mM) glucose conditions for two weeks, followed by incubation with 100 µM RG-108 for four days. Subsequently, proteins were extracted and analyzed by Western blotting. Kir4.1 protein expression was significantly elevated in astrocytes cultured under hyperglycemic conditions following RG-108 exposure compared with untreated astrocytes maintained under the same high-glucose conditions (Figure 5). Our findings demonstrate that astrocytes maintained under high-glucose conditions exhibit reduced Kir4.1 protein expression to 59.06% of controls (±6.94 SEM, n = 3); however, when we treated them with RG-108, we obtained not only a recovery of the Kir4.1 protein but an overexpression up to about 120% above the control level (Figure 5).

### 3.6. AMPK Protein Expression Is Downregulated in Astrocytes Grown in High-Glucose Conditions When Compared to Controls

**Figure 6:**
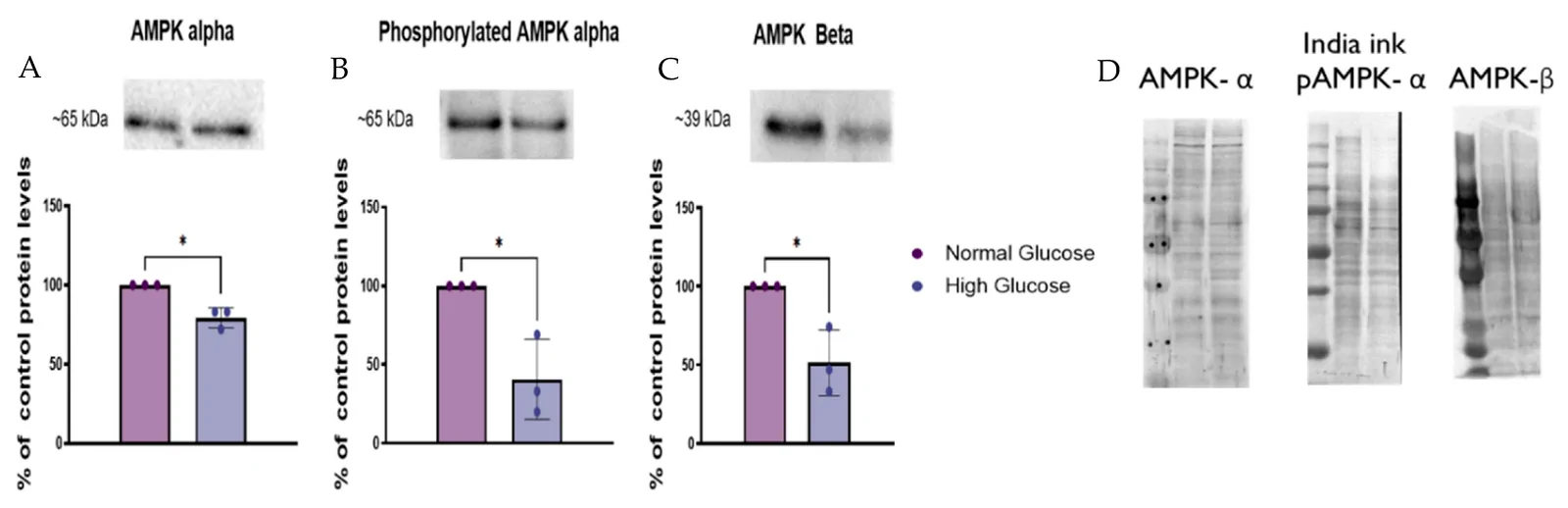
AMPK protein expression is downregulated in astrocytes grown in high-glucose conditions compared to controls. Astrocytes were cultured in normal (5 mM) and high (25 mM) glucose conditions for two weeks, followed by protein extraction for protein measurements via Western blot. (**A**). AMPK ⍺ protein was significantly decreased in astrocytes cultured in hyperglycemic conditions as compared to controls (21% of control ± 3.7 SEM, n = 3). (**B**). Phosphorylated AMPK ⍺ protein was significantly decreased in astrocytes cultured in hyperglycemic conditions as compared to controls (59.3% of control ± 14.7 SEM, n = 3). (**C**). AMPK β protein was significantly decreased in astrocytes cultured in hyperglycemic conditions as compared to controls (49% ± 12 SEM, n = 3). (**D**). pAMPKα/AMPKα ratio was 1.319 ± 0.396 in the case of low glucose and 1.075 ± 0.623 for high glucose (mean ± SEM, n = 3). In normalization from controls, the high-glucose group amounted to 81.5% of the control, which corresponds to an 18.5% decrease in the pAMPKα/AMPKα ratio. Quantification was normalized utilizing India ink (* *p* < 0.05).

Previous work has shown that regulation of AMPK can trigger a recovery of Kir4.1 in diabetic retinopathy in rat glial Müller cells [5]. To further investigate the impact of hyperglycemia on AMPK in astrocytes, cells were maintained in DMEM containing either normal (5 mM) or high (25 mM) glucose concentrations (Figure 6). Following a two-week culture period, cells were exposed to LME to eliminate microglia and obtain purified astrocyte cultures. Subsequently, cell lysates were prepared, and protein expression was assessed by Western blot analysis. Our results show that AMPK alpha (AMPK-α), phosphorylated alpha (pAMKA-α), and beta subunits (AMPK-β) are all downregulated in astrocytes cultured in high-glucose conditions when compared to controls (Figure 6). Particularly, AMPK-⍺ protein was significantly decreased in astrocytes cultured in hyperglycemic conditions as compared to controls (21% of control ± 3.7 SEM, n = 3). Phosphorylated AMPK-⍺ protein was also significantly decreased in astrocytes cultured in hyperglycemic conditions as compared to controls (59.3% of control ± 14.7 SEM, n = 3). AMPK-β protein was significantly decreased in astrocytes cultured in hyperglycemic conditions as compared to controls (49% ± 12 SEM, n = 3). Quantification was normalized utilizing India ink, * *p* < 0.05 (Figure 6).

### 3.7. Treatment with AICAR Recovers Kir4.1 Protein Expression and SIRT-1 Protein Expression in Astrocyte Culture in High-Glucose Conditions

**Figure 7:**
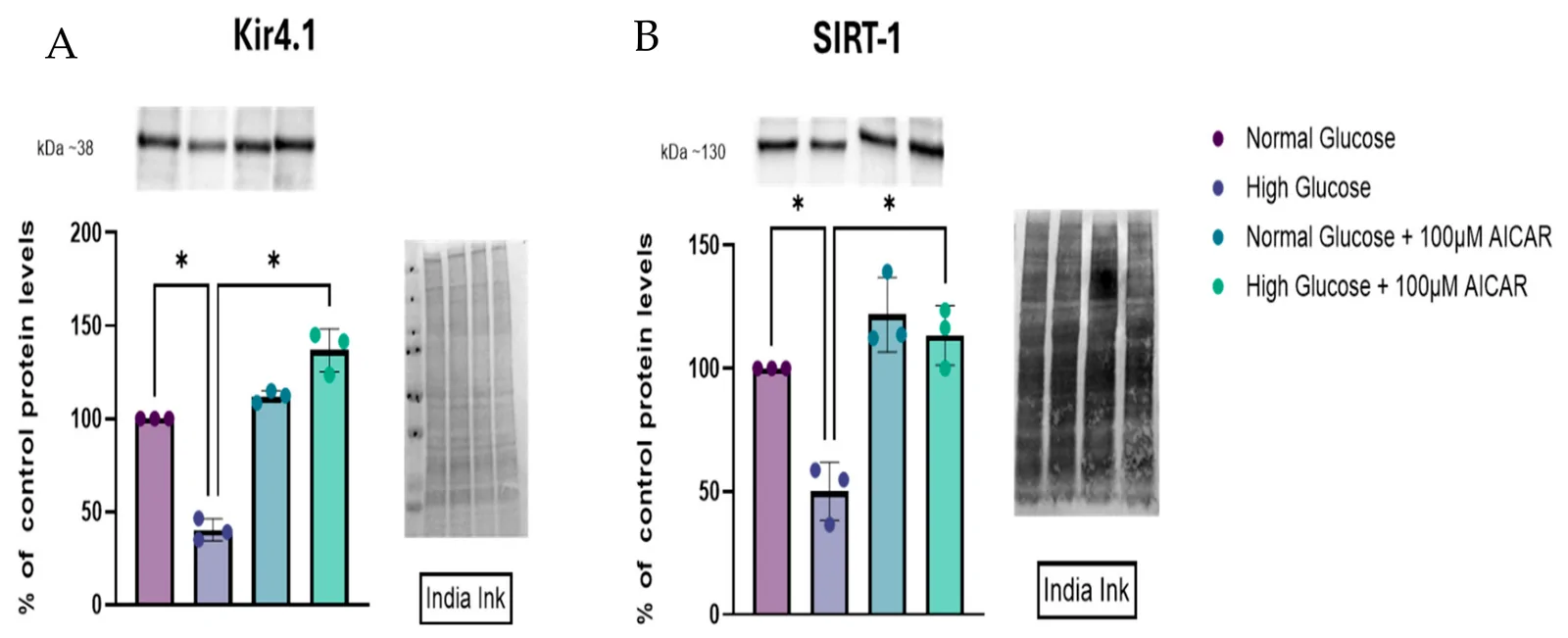
Regulation of Kir4.1 protein expression by AICAR in normal and hyperglycemic conditions. Astrocytes were cultured in normal- and high-glucose conditions for two weeks, followed by treatment with 100 µM AICAR for 24 h. (**A**). Protein extraction for protein measurements via Western blot. Kir4.1 protein expression was significantly decreased in astrocytes cultured in hyperglycemic conditions when compared to controls (59.86% of control ± 5.4 SEM, n = 3). Kir4.1 protein expression was significantly increased in astrocytes cultured in hyperglycemia when treated with AICAR compared to controls (*p* ≤ 0.000001, 95% C.I. = [−114.0 to −79.20]). Quantification was normalized utilizing India ink (* *p* < 0.05). (**B**). SIRT-1 protein expression was significantly decreased in astrocytes cultured in hyperglycemic conditions when compared to controls (49.97% of control ± 9.2 SEM, n = 3). SIRT-1 protein expression was significantly increased in astrocytes cultured in hyperglycemic conditions when treated with AICAR compared to controls (*p* = <0.000611, 95% C.I. = (−93.06 to −33.63]). Quantification was normalized utilizing India ink (* *p* < 0.05).

To further evaluate the relationship between AMPK and recovery of the Kir4.1 channel, we tested the hypothesis that treatment with 5-aminoimidazole-4-carboxamide (AICA) ribonucleotide (AICAR), a well-known and specific activator of AMPK that prevents and reverses diabetic polyneuropathy [42], would restore Kir4.1 expression in glial cells and rescue Sirtuin-1 protein (SIRT-1). SIRT-1 is a critical enzyme that uses cellular energy (via NAD^+^) to control gene silencing and activity, promoting DNA repair and reducing oxidative stress [37,40]. AMPK has been shown to activate SIRT-1 (for example, by increasing cellular NAD^+^), and SIRT-1 deacetylates upstream kinases such as LKB1, a tumor-suppressing serine/threonine kinase (STK11), thereby feeding back into the AMPK pathway and forming a regulatory loop [36]. As previously described, we prepared primary astrocyte cultures at 5 mM and 25 mM, and after two weeks, we treated them with LME and plated 300,000 cells per well in a 6-well plate. We then treated them with 100 µM AICAR and lysed the cells for Western blot analysis. Our results show that astrocytes treated with AICAR exhibit increased protein expression of Kir4.1 (Figure 7) and increased protein expression of SIRT-1 (Figure 7). Kir4.1 protein expression was significantly reduced in astrocytes maintained under hyperglycemic conditions relative to control cells (59.86% of control ± 5.4 SEM, n = 3). However, AICAR treatment significantly increased Kir4.1 protein expression in astrocytes cultured under hyperglycemic conditions compared with control cells (*p* ≤ 0.000001, 95% C.I. = [−114.0 to −79.20]). Quantification was normalized utilizing India ink (* *p* < 0.05). Hyperglycemic conditions also significantly decreased SIRT-1 protein expression in astrocytes when compared to controls (49.97% of control ± 9.2 SEM, n = 3); however, SIRT-1 protein expression was significantly increased in astrocytes cultured in hyperglycemic conditions when treated with AICAR compared to controls (*p* ≤ 0.000611, 95% C.I. = (−93.06 to −33.63]). India ink staining was employed to normalize the quantitative measurements, * *p* < 0.05 (Figure 7).

### 3.8. Astrocytes Cultured in Normal Glucose Show a Reduced Expression of Kir4.1 When Treated with an AMPK Inhibitor Known as Compound C

**Figure 8:**
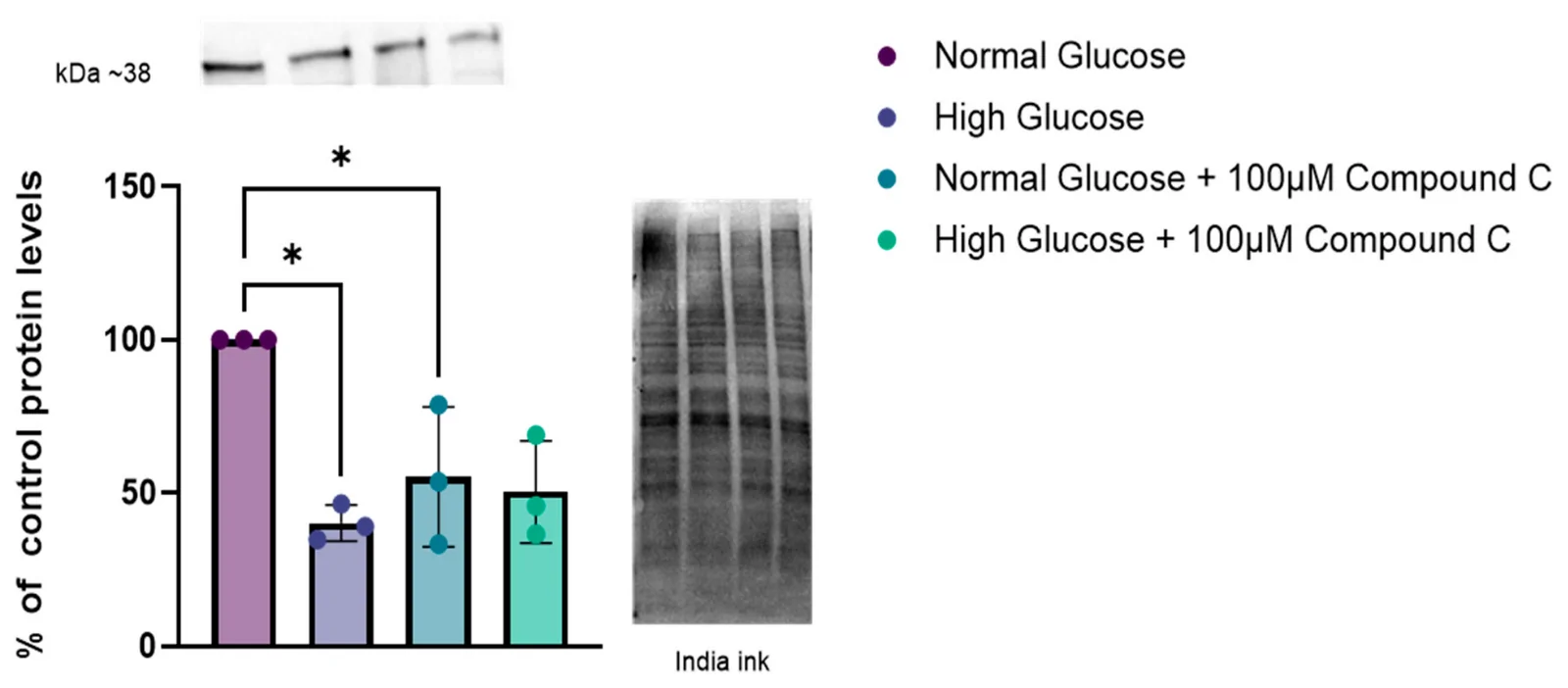
Changes in Kir4.1 protein levels in hyperglycemic conditions with and without AMPK inhibitor, compound C, normalized to normal conditions. Astrocytes were cultured in normal- and high-glucose conditions for two weeks, followed by treatment with 100µM of compound C (dorsomorphin) for 24 h. Treatment with compound C significantly reduced Kir4.1 protein expression in astrocytes maintained under normal culture conditions., when compared to controls (*p* = 0.021852, 95% C.I. = (7.029 to 82.48]). Quantification was normalized utilizing India ink (* *p* < 0.05).

Compound C (dorsomorphin) is a cell-permeable pyrazolopyrimidine used as a potent reversible and ATP-competitive inhibitor of AMPK [43]. To validate that recovery of Kir4.1 is triggered by AMPK, we tested the hypothesis that inhibition of AMPK will cause a reduction in Kir4.1 protein expression like that seen in astrocytes cultured in high-glucose conditions. As previously described, we performed a primary astrocyte culture with 5 mM and 25 mM, and after two weeks, we treated with LME and plated 300,000 cells/well in a six-well plate, where we treated with 100 µM of compound C for 24 h and lysed proteins for Western blot analysis. Changes in Kir4.1 protein levels in hyperglycemic conditions with and without AMPK inhibitor, compound C, normalized to normal conditions are shown in Figure 8. Kir4.1 protein expression was significantly decreased in astrocytes cultured in normal conditions when treated with compound C, when compared to control astrocytes (*p* = 0.021852, 95% C.I. = (7.029 to 82.48]). India ink staining was employed to normalize the quantitative measurements (* *p* < 0.05). Compound C causes a decrease in the Kir4.1 protein expression in astrocytes cultured in normal glucose (Figure 8).

## 4. Discussion

In the current work, we highlighted key proteins being affected by high glucose levels, including upregulation of H1.0, glial maturation factors, histones, and glyceraldehyde-3-phosphate dehydrogenase (Figure 1), consistent with metabolic and epigenetic remodeling under diabetic conditions [44,45]. We also observed downregulation of potassium channels, such as Kir4.1 (Figure 3, Figure 5, Figure 7 and Figure 8), splicing factors, histone-arginine methyltransferase, glutamate receptors, and AMPK β-subunit (Figure 1), consistent with impaired metabolic sensing and ion homeostasis pathways in astrocytes [21,46,47]. These results suggest a link between hyperglycemia and regulation of Kir4.1 potassium channels via metabolic regulators and DNA methylation mechanisms [21,35]. Our analysis of the TMT-proteomic data showed specifically that diabetic mice have reduced protein expression of key metabolic proteins involved in maintaining astrocyte function. Particularly, we found that hyperglycemia (25 mM glucose) (i) increased DNMT-1 protein levels in astrocytes and (ii) decreased AMPK and (iii) SIRT-1 levels, accompanied by a reduction in Kir4.1 protein expression. Pharmacological inhibition of DNMT-1 with RG-108 decreased DNMT-1 levels and restored Kir4.1. Treatment with AICAR, a selective AMPK activator, rescued SIRT-1 and Kir4.1 expression. Therefore, hyperglycemia engages dual regulatory pathways—(i) epigenetic via DNMT-1 and (ii) metabolic via AMPK/SIRT-1— which converge in Kir4.1 downregulation in astrocytes. We concluded that both DNMT-1 inhibition and AMPK activation rescued Kir4.1 expression, suggesting functional support for potassium and glutamate homeostasis and possible pharmacological interventions.

Previous studies have established that DNA methylation directly influences KCNJ10 gene expression and Kir4.1 protein levels following injury in the central nervous system (CNS) in the hippocampus as well as in the peripheral nervous system (PNS) in the spinal cord [21,35,41]. However, this has not been fully established in astrocytes under hyperglycemic conditions. We investigated DNA methylation of *CpG* islands in astrocytes cultured under high-glucose conditions compared to control conditions. Our methylation analysis showed bidirectional methylation changes across multiple genes and pathways (Figure 2), consistent with metabolic stress–induced epigenetic remodeling [44]. Prior findings by Olsen and colleagues demonstrated DNMT1-dependent methylation of KCNJ10 in pathological conditions [35], although direct validation in rat astrocytes remains limited due to species-specific primer constraints. Our results are consistent with these findings, as other Kir channel family genes also showed hypermethylation patterns, suggesting broader epigenetic regulation of potassium channels under hyperglycemic stress (Figure 2). Taken together, these findings support a role for DNA methylation in regulating Kir channel expression in astrocytes.

We further evaluated DNMT-1 expression and activity in astrocytes cultured under high-glucose conditions (Figure 3 and Figure 4). Western blot analysis showed increased DNMT-1 protein expression, consistent with enhanced methylation capacity under metabolic stress [35,48]. DNMT-1 activity assays further confirmed increased methyltransferase activity, consistent with pyrosequencing data and prior reports linking metabolic stress to epigenetic remodeling [44,45]. Pharmacological inhibition of DNMT-1 with RG-108 partially restored Kir4.1 protein expression under high-glucose conditions (Figure 4), supporting a functional role for DNA methylation in Kir4.1 regulation [21,35].

Taken together, these results support DNA methylation as a key mechanism underlying reduced Kir4.1 expression in astrocytes under hyperglycemic conditions. Increased DNMT1 expression and activity, combined with decreased Kir4.1 expression and partial rescue following DNMT-1 inhibition, suggest that hyperglycemia promotes epigenetic repression of KCNJ10 and related potassium channel genes [1,21].

Regulation of DNA methylation pathways can also be modulated by AMPK, a critical metabolic sensor linking energy status to epigenetic regulation [49,50]. AMPK regulates DNMT-1, DNMT-3a, and DNMT-3b activity in response to metabolic stress [47,50]. Our proteomic data show downregulation of AMPK β-subunit in diabetic mice (Figure 1), consistent with impaired metabolic sensing. Western blot results further show reduced AMPK α and β subunits, as well as decreased phosphorylated AMPK α under high-glucose conditions, consistent with suppressed AMPK signaling [49,51].

Recent studies have shown that metabolic pathways regulate Kir4.1 [3]. Astrocytes rely highly on glycolysis (TCA Krebs cycle) for energy. The side product of glycolysis, methylglyoxal (MG), is very toxic; glyoxalases inactivate such toxicity, catalyzing glutathione (GSH) to convert MG into safe and useful D-lactate. When hyperglycemia causes a decrease in glyoxalase (Glo1 and Glo2) contents and Kir4.1 protein expression level decreases [3], aminoguanidine treatment rescues the expression and function of Glo1, Glo2, and Kir4.1 channels in astrocytes [3]. The metabolic pathway via AMPK we describe here, activation of AMPK using 5-aminoimidazole-4-carboxamide (AICA) ribonucleotide (AICAR), a well-known and specific activator of AMPK, restored Kir4.1 protein levels and SIRT1 protein expression (Figure 7). Alternatively, the inhibition of AMPK by compound C produces Kir4.1 downregulation (Figure 8). These findings are consistent with observations that AMPK activation protects against metabolic decline, and AMPK activation increases glycolysis [46,47]. Conversely, AMPK inhibition with compound C reduced Kir4.1 expression even under normal glucose (Figure 8).

Taking these results together, we can show DNA methylation as a possible pathway in the reduced expression of the Kir4.1 protein in hyperglycemic astrocytes. High glucose affects the Kir4.1 expression and increases DNMT-1 activity, which ties it directly to the regulation of Kir4.1 via DNA methylation of the KCNJ10 gene by DNMT-1.

Regulation of the DNA methylation pathway can be achieved through AMPK, a critical metabolic sensor. Other groups have shown that AMPK can directly modulate DNA methylation via DNMT-1, DNMT-3a, and DNMT-3b. Our proteomic analysis shows that the AMPK beta subunit is downregulated in diabetic mice (Figure 1). Our Western blot analysis revealed reduced expression of the AMPK α and β subunits in astrocytes maintained under high-glucose conditions, accompanied by decreased levels of phosphorylated AMPK α, the activated form of the α subunit. With this information, we can deduce that AMPK is a key player in the regulation of Kir4.1 via multiple pathways, one of which may be DNA methylation of KCNJ10 via DNMT-1. Taking this into consideration, we evaluated whether AMPK could trigger a recovery of Kir4.1 in astrocytes, as shown in Müller cells [5]. Through the use of AICAR, a specific AMPK activator, we demonstrated the restoration of Kir4.1 and SIRT-1 protein expression in astrocytes maintained under high-glucose conditions relative to control cells (Figure 7). To further validate that AMPK is responsible for this upregulation of Kir4.1, we inhibited the activation of AMPK using compound C, a known inhibitor of AMPK, which caused a reduction in the protein expression of Kir4.1 in astrocytes grown in normal glucose when compared to controls (Figure 8). This reduction is like the reduction in Kir4.1 shown in high-glucose conditions, leading us to conclude that AMPK is, in fact, a key metabolic regulator for Kir4.1 protein expression and possibly of its function.

SAM (Scheme 1) participates not only in the methylation of DNA, but when decarboxylated to dcSAM by the enzyme S-adenosylmethionine decarboxylase (AdometDC), acts as an aminopropyl group donor during the synthesis of PAs putrescine, spermidine, and spermine. PAs play role in K-current regulation [19,20,22,23,52]. Intriguingly, overexpression of the catabolizing polyamine spermine (SPM) enzyme, spermine oxidase (SMOX), triggers an astrocytic reactive response [53] and multiple degenerative characteristics in the CNS [54]. Recently, attention has turned to PAs such as agmatine (AGM) and spermidine (SPD); AGM contains a guanidine group similar to aminoguanidine (AG) and metformin, drugs that protect against hyperglycemia [3,5], and agmatine is very effective in the treatment of psychiatric disorders [55,56,57]. SPD is also involved in neuroprotection and longevity [58,59,60,61,62].

Therefore, elevated SAM levels are closely linked to both (i) DNA methylation and (ii) PA synthesis; SAM boosts DNMT activity, and dcSAM boosts PA synthesis. The rate of SAM conversion into dcSAM shifts the balance between active methylation and its inhibition. Consequently, SAM and PAs play key roles (i) in Kir4.1 channel expression and (ii) in Kir4.1 channel conductance rectification by PAs. DNMT-1 maintains methylation during DNA replication and differentiation, and its misregulation is linked to abnormal gene silencing in neurological and metabolic diseases.

## 5. Conclusions

We conclude that primary astrocytes from rats exhibit compromised Kir4.1 potassium channel expression during hyperglycemia, correlating with alterations in DNMT-1 expression and triggering a hypermethylated state of the KCNJ10 gene due to uncontrolled DNMT-1 activity under high-glucose conditions. These effects are reversible with (i) inhibition of DNMT-1 using the specific inhibitor RG-108 or (ii) activation of AMPK using AICAR, a specific activator of the AMPK/SIRT-1 pathway. Our graphic abstract (Scheme 1) illustrates our findings. Further studies are required to validate whether recovery of Kir4.1 protein expression can also restore astrocyte potassium buffering and glutamate clearance. Taking in account the importance of Kir4.1 channels in the function of oligodendrocytes [63,64,65], Müller glial cells [4,5,20,28], satellite glial cells [66,67,68], pericytes [69,70], Bergmann glia [71,72], ependyma [73], and astrocytes [6,7,11,74] with sex dependence [7], our findings may offer important insights in the study of hyperglycemia-induced downregulation of Kir4.1 channels and Cx43 GJs [75] in different glial cells.

## Data Availability

The original contributions presented in this study are included in the article. Further inquiries can be directed to the corresponding authors.

## Abbreviations

The following abbreviations are used in this manuscript:

Adomet	Adenosylmethionine
AICAR	5-aminoimidazole-4-carboxamide ribonucleoside, a specific activator of AMPK
AMPK	Adenylate Charge-Regulated Protein Kinase, also called 5′-adenosine monophosphate (AMP)-activated protein kinase
ANOVA	Analysis of variance
CNS	Central nervous system
*CpG*	Cytosine-phosphate-Guanine island (cytosine (C) is linked to a guanine (G) by a phosphate group in the 5′ → 3′ direction).
DB/DB	Homozygous diabetic mouse carrying the leptin-receptor mutation
DB/+	Heterozygous non-diabetic control mouse
dcSAM	decarboxylated S-adenosylmethionine
DMEM	Dulbecco’s modified Eagle medium for cell cultures
DMR	Differentially methylated region
DNA	Deoxyribonucleic acid
DNMT1	DNA methyltransferase-1
EDTA	Ethylenediaminetetraacetic acid
gDNA	genomic DNA (a complete set of chromosomal genetic material from astrocytes)
IACUC	Institutional Animal Care and Use Committee
I/V	Current–voltage relationship
KCl	Potassium chloride
KCNJ10	Gene encoding Kir4.1
Kir4.1	Inwardly rectifying potassium channel subunit 4.1
LME	L-Leucine methyl ester
mRNA	Messenger ribonucleic acid
PBS	Phosphate-buffered saline
PMSF	Phenylmethylsulphonyl fluoride lysis buffer
PNS	Peripheral nervous system
qPCR	Quantitative polymerase chain reaction
RG-108	A specific DNA methyltransferase inhibitor used to inactivate DNMT1
RIPA	Radioimmunoprecipitation assay buffer
RNA	Ribonucleic acid
RT-qPCR	Reverse transcription quantitative PCR
SAM	S-adenosylmethionine
SDS-PAGE	Sodium dodecyl sulfate–polyacrylamide gel electrophoresis
SEM	Standard error of the mean
SIRT-1	Sirtuin-1, a critical enzyme that uses cellular energy via NAD^+^
Tris-HCl	Tris(hydroxymethyl)aminomethane hydrochloride
TRFC	Transferrin receptor gene (housekeeping gene used in qPCR)
WB	Western blot
